# SFTPB Expression Predicts Favorable Survival in Lung Adenocarcinoma but Poor Prognosis in Lung Squamous Cell Carcinoma

**DOI:** 10.3390/medicina62061140

**Published:** 2026-06-11

**Authors:** Soonsoo Kim, Hyowon Hong, Jae-Ho Lee

**Affiliations:** 1 Medical Course, School of Medicine, Keimyung University, Daegu 42601, Republic of Korea; 2 Department of Anatomy, School of Medicine, Keimyung University, Daegu 42601, Republic of Korea

**Keywords:** SFTPB, lung cancer, non-small cell lung cancer (NSCLC), adenocarcinoma (AD), squamous cell carcinoma (SCC)

## Abstract

*Background and Objectives*: Surfactant protein B (SFTPB) is a surfactant-associated protein secreted by alveolar type II epithelial cells that plays a critical role in maintaining alveolar stability and surface tension. Although SFTPB is closely associated with pulmonary epithelial differentiation, its clinical significance in different non-small cell lung cancer (NSCLC) subtypes remains unclear. This study investigated the clinicopathologic and prognostic significance of SFTPB expression in lung adenocarcinoma (AD) and lung squamous cell carcinoma (SCC) using The Cancer Genome Atlas (TCGA) dataset. *Materials and Methods*: SFTPB mRNA expression data and clinicopathologic information were obtained from TCGA cohorts of AD and SCC patients. Patients were stratified into high- and low-expression groups according to median SFTPB expression levels. Associations between SFTPB expression and clinicopathologic variables were analyzed, and correlation analyses were performed with major oncogenic genes. Overall survival (OS) and relapse-free survival (RFS) were evaluated using Kaplan–Meier survival analysis and log-rank testing. Multivariate Cox proportional hazards regression analyses were performed after adjustment for age, sex, and pathological stage. *Results*: In AD, high SFTPB expression was significantly associated with lower pathologic stage (*p* = 0.011) and lower N stage (*p* = 0.006). SFTPB expression showed significant negative correlations with EGFR (R = −0.140, *p* = 0.002) and BRAF (R = −0.177, *p* < 0.001) and a positive correlation with TP53 (R = 0.128, *p* = 0.004). Patients with high SFTPB expression demonstrated significantly improved OS compared with those with low expression (*p* < 0.001)**,** while a trend toward prolonged RFS was observed without statistical significance (*p* = 0.089). Multivariate analysis confirmed high SFTPB expression as an independent favorable prognostic factor in AD (HR = 0.551, 95% CI = 0.405–0.748, *p* < 0.001). In SCC, high SFTPB expression was also significantly associated with lower pathologic stage (*p* = 0.009) and lower N stage (*p* = 0.007). SFTPB expression showed significant negative correlations with SOX2 (R = −0.176, *p* < 0.001), PIK3CA (R = −0.143, *p* = 0.002), and TP53 (R = −0.101, *p* = 0.026). In contrast to AD, high SFTPB expression was significantly associated with poorer OS (*p* = 0.026), whereas no significant difference in RFS was observed (*p* = 0.307). Multivariate analysis demonstrated that high SFTPB expression was an independent adverse prognostic factor in SCC (HR = 1.347, 95% CI = 1.028–1.767, *p* = 0.031). *Conclusions*: SFTPB expression is significantly associated with clinicopathologic characteristics and molecular signatures in both AD and SCC. However, its prognostic implications differ according to histologic subtype. High SFTPB expression independently predicts favorable survival in AD but unfavorable survival in SCC, suggesting distinct lineage-specific biological roles in NSCLC. These findings support SFTPB as a subtype-specific prognostic biomarker reflecting differential differentiation states and lineage context in NSCLC.

## 1. Introduction

Lung cancer remains the leading cause of cancer-related mortality worldwide, with over 2 million new cases and nearly 2 million deaths annually [1]. Non-small cell lung cancer (NSCLC), accounting for approximately 85% of all lung cancers, is mainly classified into lung adenocarcinoma (AD) and lung squamous cell carcinoma (SCC) [2,3,4]. Although both are categorized under NSCLC, growing genomic and transcriptomic evidence has established that AD and SCC are biologically distinct entities with different genomic landscapes and clinical behaviors [5,6,7,8,9].

AD is frequently driven by oncogenic alterations in EGFR, KRAS, BRAF, and ALK, whereas SCC is characterized by recurrent alterations in TP53, CDKN2A, SOX2, PIK3CA, and NOTCH1 [10,11,12,13,14]. Given these distinct molecular backgrounds, subtype-specific biomarkers are necessary to better understand tumor biology, refine prognostic stratification, and potentially guide subtype-specific therapeutic strategies.

Surfactant protein B (SFTPB) encodes a critical hydrophobic surfactant-associated protein that plays an essential role in pulmonary surfactant function and alveolar stability [15,16]. SFTPB is predominantly expressed in type II alveolar epithelial cells, which are considered one of the cells of origin for lung adenocarcinoma [17]. Beyond its physiological role in maintaining lung homeostasis, dysregulation of surfactant-associated genes has been linked to tumor differentiation, lineage identity, and clinical heterogeneity in lung cancer [18,19]. Previous studies have suggested that surfactant protein expression may reflect tumor differentiation and cellular origin in AD, whereas such expression is generally absent or reduced in SCC due to its squamous lineage derivation [17,18,19]. Consequently, the biological and clinical implications of SFTPB expression may differ substantially according to histologic subtype and lineage context.

However, despite its biological relevance, the clinicopathologic and prognostic significance of SFTPB expression across AD and SCC has not been systematically investigated. Considering the lineage specificity of surfactant proteins, we hypothesized that SFTPB expression may demonstrate subtype-dependent clinical and molecular associations in NSCLC. Accordingly, we performed an integrated TCGA-based analysis to characterize SFTPB expression in AD and SCC, explore its associations with clinicopathologic characteristics and key oncogenic pathways, and evaluate its subtype-specific prognostic significance using survival and multivariate prognostic analyses.

## 2. Materials and Methods

### 2.1. TCGA Data Analysis

**Figure 1:**
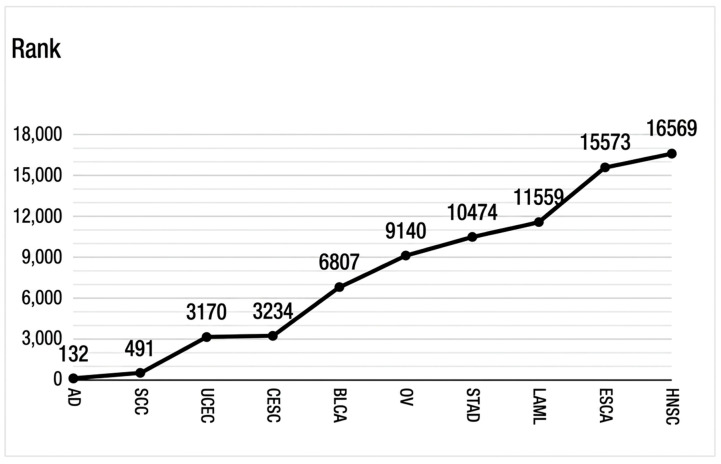
Pan-cancer ranking of the prognostic significance of SFTPB expression across TCGA cancer types. Cancer types were ranked according to the statistical significance of the association between SFTPB expression and overall survival. Lung adenocarcinoma (AD) and lung squamous cell carcinoma (SCC) showed the highest prognostic relevance among the analyzed cancer types and were therefore selected for subsequent detailed clinicopathological and survival analyses. UCEC, uterine corpus endometrial carcinoma; CESC, cervical squamous cell carcinoma and endocervical adenocarcinoma; BLCA, bladder urothelial cancer; OV, ovarian serous cystadenocarcinoma; STAD, stomach adenocarcinoma; LAML, acute myeloid leukemia; ESCA, esophageal carcinoma; HNSC, head and neck squamous cell carcinoma.

We utilized primary data from The Cancer Genome Atlas (TCGA) portal (http://cancergenome.nih.gov/) (accessed on 14 October 2025). The data provided *p*-value rankings for the prognostic significance of SFTPB expression across various cancer types (Figure 1). Among these, AD and SCC demonstrated the most significant associations and were therefore selected for further detailed analysis. A total of 980 NSCLC patients including 492 patients with AD and 488 with SCC were included in the clinical and survival analyses. mRNA expression profiles and corresponding clinical information were downloaded from the TCGA database. Clinical variables included age, sex, pathologic TNM stage, EGFR mutation status, smoking status, and survival information. RNA sequencing expression data were generated using the TCGA RNA Seq V2 pipeline and normalized using the RSEM (RNA-Seq by Expectation Maximization) method as provided by cBioPortal. Patients lacking survival information were excluded from survival analyses. Cases with missing clinicopathological variables were excluded from the corresponding subgroup analyses. This study met the publication guidelines for using TCGA data sets (https://portal.gdc.cancer.gov/ accessed on 14 October 2025).

### 2.2. Statistical Analysis

Data were analyzed using SPSS software (version 25.0; IBM SPSS, Armonk, NY, USA). For statistical analyses, patients were stratified into high- and low-expression groups according to the median SFTPB mRNA expression level within each histologic subtype. Tumor staging was determined according to the seventh edition of the American Joint Committee on Cancer (AJCC) staging system. Clinicopathological variables (including age, sex, pathological TNM stage, EGFR mutation, and smoking status) were analyzed using the chi-square test. Spearman’s correlation coefficient was used to assess correlations between SFTPB expression and other genes associated with AD or SCC. Overall survival (OS) and relapse-free survival (RFS) were evaluated using Kaplan–Meier survival analysis and compared using the log-rank test. OS was defined as the time from diagnosis to death from any cause, whereas RFS was defined as the time from diagnosis to the first documented recurrence or progression event. To evaluate the independent prognostic significance of SFTPB expression, multivariate Cox proportional hazards regression analyses were performed separately for the AD and SCC cohorts. Age at diagnosis, sex, pathological stage, and SFTPB expression status were included as covariates in the multivariate models. Hazard ratios (HRs) and 95% confidence intervals (CIs) were calculated. A *p*-value of <0.05 was considered statistically significant.

## 3. Results

To elucidate the association between SFTPB expression and clinicopathologic characteristics, patients were stratified into high- and low-expression groups based on the median SFTPB mRNA level. The relationships between SFTPB expression and clinical variables were then analyzed in both AD and SCC cohorts.

In AD, higher SFTPB expression was significantly associated with lower pathologic stage (*p* = 0.011) and lower N stage (*p* = 0.006; Table 1). Similarly, in SCC, higher SFTPB expression was significantly associated with both lower pathologic stage (*p* = 0.009) and lower N stage (*p* = 0.007), suggesting that elevated SFTPB expression is linked to less advanced disease in both subtypes.

Correlation analysis was performed to evaluate the relationship between SFTPB expression and key oncogenic drivers (EGFR, KRAS, TP53, ALK, and BRAF), as well as age, in AD (Table 2). SFTPB expression showed significant negative correlations with EGFR (R = −0.140, *p* = 0.002) and BRAF (R = −0.177, *p* < 0.001), while a positive correlation was observed with TP53 (R = 0.128, *p* = 0.004). A weak inverse correlation with KRAS expression was observed; however, this association did not reach statistical significance (R = −0.076, *p* = 0.090).

In SCC, correlation analysis revealed that SFTPB expression was significantly negatively correlated with SOX2 (R = −0.176, *p* < 0.001), PIK3CA (R = −0.143, *p* = 0.002), and TP53 (R = −0.101, *p* = 0.026) (Table 3). A weak negative correlation with CDKN2A expression was observed, although this did not reach statistical significance (R = −0.085, *p* = 0.062).

**Figure 2:**
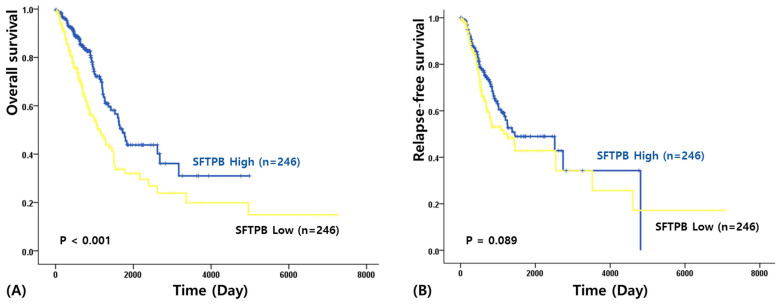
Overall survival (**A**) and relapse-free survival (**B**) analysis in lung adenocarcinoma.

Overall survival (OS) and relapse-free survival (RFS) were evaluated using Kaplan–Meier survival analysis and compared by the log-rank test. In lung adenocarcinoma (AD), patients with high SFTPB expression exhibited significantly better OS than those with low SFTPB expression (1778.00 ± 50.59 days vs. 1147.00 ± 60.96 days, χ^2^ = 18.280, *p* < 0.001; Figure 2A). Similarly, the high-expression group demonstrated a longer median RFS than the low-expression group (1450.00 ± 536.97 days vs. 1258.00 ± 246.48 days); however, this difference did not reach statistical significance (χ^2^ = 2.887, *p* = 0.089; Figure 2B). These findings indicate that elevated SFTPB expression is strongly associated with favorable overall survival and may also be linked to prolonged relapse-free survival in AD.

**Figure 3:**
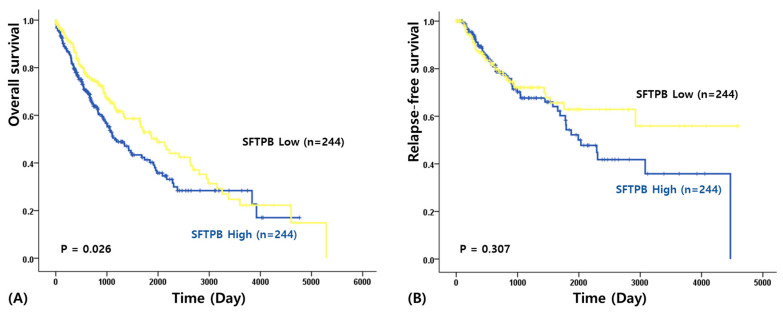
Overall survival (**A**) and relapse-free survival (**B**) analysis in lung squamous cell carcinoma.

In SCC, the median survival was 1655.00 ± 135.21 days. High SFTPB expression was significantly associated with poorer OS. Patients with high SFTPB expression consistently exhibited shorter survival compared to those with low SFTPB expression (1154.00 ± 118.42 vs. 1984.00 ± 153.28 days, χ^2^ = 5.006, *p* < 0.05; Figure 3A). For RFS, the high-expression group also showed a shorter median relapse-free survival than the low-expression group (2033 days vs. not reached); however, the difference was not statistically significant (χ^2^ = 1.043, *p* = 0.307; Figure 3B). The median RFS was not reached in the low-expression group because fewer than 50% of patients experienced recurrence during the follow-up period. These findings indicate that the prognostic impact of SFTPB differs between AD and SCC, suggesting subtype-specific biological roles.

To further evaluate the prognostic significance of SFTPB, multivariate Cox proportional hazards regression analyses were performed after adjustment for age, sex, and pathological stage (Table 4). In AD, high SFTPB expression remained an independent favorable prognostic factor for overall survival (HR = 0.551, 95% CI = 0.405–0.748, *p* < 0.001). Advanced pathological stage was also independently associated with poorer survival (HR = 2.247, 95% CI = 1.641–3.077, *p* < 0.001), whereas age and sex were not significant predictors. In contrast, high SFTPB expression was independently associated with worse overall survival in SCC (HR = 1.347, 95% CI = 1.028–1.767, *p* = 0.031). Advanced pathological stage remained a significant adverse prognostic factor (HR = 1.574, 95% CI = 1.143–2.168, *p* = 0.005), and increasing age was also associated with poorer survival (HR = 1.017, 95% CI = 1.001–1.035, *p* = 0.042). These findings demonstrate that SFTPB exerts opposite prognostic effects in the two major non-small cell lung cancer subtypes, acting as a favorable prognostic marker in AD but an unfavorable prognostic marker in SCC.

## 4. Discussion

In this study, we investigated the expression and clinical significance of SFTPB in AD and SCC using TCGA datasets and identified distinct subtype-specific associations with tumor progression and prognosis. Although higher SFTPB expression was associated with earlier pathologic stage and lower nodal status in both AD and SCC, its prognostic impact differed markedly between the two histologic subtypes, suggesting fundamentally different biological roles according to tumor lineage context.

In AD, high SFTPB expression was significantly associated with lower pathologic stage and lower N stage. Patients in the high-expression group were more frequently classified as stage I and N0, whereas advanced-stage tumors more commonly exhibited low SFTPB expression. These findings suggest that loss of SFTPB expression is associated with tumor progression and may be linked to biological processes related to metastatic dissemination in AD. Since SFTPB is a surfactant protein produced by alveolar type II epithelial cells, which are considered a major cell of origin for AD, its expression likely reflects preservation of alveolar lineage differentiation [20,21]. Maintenance of surfactant homeostasis may contribute to alveolar structural integrity and reduce tumor cell detachment and invasion, thereby limiting metastatic spread.

Previous studies have demonstrated that surfactant-associated proteins are preferentially expressed in well-differentiated adenocarcinomas and tend to decrease during tumor dedifferentiation and progression [15,16,17,18,19,20,21]. Our findings are consistent with these observations and further support the concept that SFTPB functions as a differentiation-associated marker in AD. From a biological perspective, preserved alveolar differentiation may antagonize aggressive oncogenic programs and maintain a less invasive tumor phenotype.

Correlation analysis in AD further supported this interpretation. SFTPB expression showed significant negative correlations with EGFR and BRAF while exhibiting a positive correlation with TP53. Although the correlation coefficients were modest, indicating relatively weak associations at the individual gene level, the observed patterns were biologically consistent with the clinicopathological and survival findings. Such weak correlations are not unexpected given the multifactorial nature of tumor biology, in which individual genes rarely exert dominant effects on clinical behavior. Therefore, these correlations should not be interpreted as evidence of strong regulatory relationships but rather as supportive observations suggesting that tumors with higher SFTPB expression may possess relatively attenuated oncogenic signaling and partially preserved tumor suppressive activity. In particular, EGFR and BRAF activation are strongly associated with proliferative signaling and tumor progression, whereas preservation of lineage differentiation programs may counterbalance these oncogenic pathways. Consistent with this interpretation, patients with high SFTPB expression demonstrated significantly improved overall survival, supporting the notion that SFTPB expression reflects a more differentiated and biologically less aggressive subtype of AD. Importantly, the favorable prognostic impact of SFTPB remained significant after adjustment for age, sex, and pathological stage in multivariate Cox regression analysis, indicating that SFTPB provides prognostic information beyond conventional clinicopathologic factors.

The divergent prognostic effects of SFTPB between AD and SCC may be explained, at least in part, by their distinct cells of origin and lineage-specific differentiation programs. Lung adenocarcinoma is generally thought to arise from distal airway epithelial cells, particularly alveolar type II pneumocytes, which physiologically express surfactant-associated proteins including SFTPB. Consequently, preservation of SFTPB expression in AD likely reflects maintenance of normal alveolar differentiation and lineage fidelity. In contrast, squamous cell carcinoma develops through a distinct pathogenic pathway involving bronchial basal epithelial cells and squamous metaplasia, cellular populations that normally do not express SFTPB. Therefore, SFTPB expression in SCC may represent aberrant activation of non-squamous lineage programs rather than preservation of normal cellular identity. This fundamental biological distinction may underlie the opposite prognostic associations observed between the two NSCLC subtypes.

In contrast, the biological implications of SFTPB expression in SCC appear to be substantially different. Although higher SFTPB expression was also associated with earlier pathologic stage and lower N stage in SCC, patients with high SFTPB expression paradoxically demonstrated significantly worse overall survival. Unlike AD, SFTPB is not a canonical marker of squamous differentiation, and therefore its expression in SCC may reflect aberrant lineage programs rather than preservation of normal cellular identity.

Correlation analysis in SCC revealed significant negative associations between SFTPB and SOX2 and PIK3CA, both of which are closely linked to canonical squamous oncogenic signaling through chromosome 3q amplification. Although the correlation coefficients were relatively small, the consistent negative associations with SOX2 and PIK3CA suggest a potential relationship between SFTPB expression and reduced squamous lineage commitment. Given the weak magnitude of these correlations, their clinical significance should be interpreted cautiously and viewed primarily as supportive evidence for broader biological trends rather than direct mechanistic interactions. One possible explanation is that ectopic or residual SFTPB expression reflects lineage plasticity or transdifferentiation states within SCC. Increasing evidence suggests that lineage plasticity contributes to intratumoral heterogeneity, therapeutic resistance, and aggressive clinical behavior across multiple cancer types [22,23]. Therefore, while SFTPB expression in AD may indicate preserved alveolar differentiation and favorable biology, its expression in SCC may instead represent aberrant differentiation states associated with biologic instability and poor prognosis. Consistent with these observations, high SFTPB expression remained independently associated with worse overall survival in multivariate analysis, further supporting its biological relevance in SCC beyond its association with pathological stage.

Interestingly, although SFTPB expression was significantly associated with overall survival in both AD and SCC, its association with relapse-free survival did not reach statistical significance in either subtype. In AD, patients with high SFTPB expression exhibited a trend toward prolonged relapse-free survival, whereas in SCC the low-expression group demonstrated a longer relapse-free survival despite the absence of statistical significance. These findings suggest that the prognostic value of SFTPB may extend beyond recurrence-related events and could reflect biological factors influencing long-term disease progression, treatment response, or survival following recurrence. Alternatively, the relatively limited number of recurrence events within TCGA cohorts may have reduced the statistical power to detect significant differences in relapse-free survival.

Overall, these findings suggest that the prognostic significance of SFTPB is highly dependent on histologic subtype and lineage context. In AD, high SFTPB expression was associated with earlier-stage disease, N0 status, and improved overall survival, supporting the interpretation that preservation of alveolar differentiation is linked to a less aggressive tumor phenotype. Given the physiologic role of SFTPB in maintaining surfactant homeostasis and alveolar integrity, its expression may reflect biological processes associated with preserved epithelial integrity and reduced metastatic potential. However, the present study did not directly investigate the mechanistic relationship between SFTPB expression and tumor invasion or metastatic dissemination, and therefore these interpretations should be regarded as biologically plausible hypotheses rather than direct mechanistic conclusions.

In contrast, the prognostic implications of SFTPB expression in SCC appear to differ substantially. Although higher SFTPB expression was more frequently observed in earlier-stage tumors, its association with survival was unfavorable. Because SFTPB is not a canonical marker of squamous lineage differentiation, its expression in SCC may reflect disrupted lineage fidelity, aberrant differentiation programs, or increased cellular plasticity rather than preservation of normal epithelial identity.

From a broader biological perspective, the prognostic impact of lineage-associated markers may depend not only on their absolute expression levels but also on the cellular context in which they are expressed. In AD, SFTPB expression is concordant with the physiologic differentiation program of alveolar epithelial cells and may therefore indicate preservation of normal lineage characteristics. In SCC, however, expression of an alveolar lineage marker may signify lineage ambiguity or reprogramming, biological processes increasingly recognized as contributors to tumor heterogeneity and adverse clinical outcomes.

From a broader biological perspective, maintenance of alveolar differentiation programs may be associated with preservation of epithelial integrity and suppressing epithelial–mesenchymal transition (EMT), whereas loss of SFTPB expression during tumor progression may indicate dedifferentiation and acquisition of more aggressive phenotypes [22,23]. Because the present study was based on transcriptomic and clinical correlation analyses, these interpretations should be regarded as biologically plausible hypotheses rather than direct mechanistic conclusions. Conversely, ectopic SFTPB expression in SCC may represent lineage instability associated with unfavorable tumor behavior. Such lineage instability has been linked to enhanced tumor adaptability, intratumoral heterogeneity, and therapeutic resistance in multiple malignancies. Therefore, the adverse prognostic impact of SFTPB in SCC may not necessarily reflect a direct biological effect of the protein itself but rather its role as a surrogate marker of aberrant differentiation states and lineage reprogramming. Collectively, these findings support the concept that SFTPB functions not simply as a structural surfactant protein but also as a lineage-associated biomarker reflecting distinct differentiation states in NSCLC.

## 5. Conclusions

Several limitations should be acknowledged. First, this study was retrospective in nature and based primarily on transcriptomic datasets, which precludes direct inference of causality. Second, protein-level validation using immunohistochemistry or functional assays was not performed. Therefore, additional experimental studies are necessary to determine whether SFTPB directly modulates tumor progression or primarily serves as a marker of cellular differentiation status. In particular, the potential roles of SFTPB in tumor invasion, metastatic dissemination, and lineage plasticity require further mechanistic investigation. Third, recurrence information was incomplete for a subset of TCGA patients, which may have limited the statistical power of the relapse-free survival analyses. Finally, tumor microenvironmental influences and intratumoral heterogeneity were not directly evaluated and warrant further investigation.

## Figures and Tables

**Table 1:** Clinical characteristics of SFTPB expression in adenocarcinoma and squamous cell carcinoma.

	AD			SCC		
	High	Low	*p*-Value	High	Low	*p*-Value
Age						
≤65	110	126	0.148	86	103	0.122
>65	135	119		157	141	
Gender						
Female	133	133	1	70	56	0.140
Male	112	112		173	188	
Smoking status						
Yes	165	168	0.772	212	200	0.134
No	81	78		32	44	
Pathologic stage						
Stage I	149	113	0.011	134	103	0.009
Stage II	49	68		61	95	
Stage III	34	45		42	41	
Stage IV	9	15		4	3	
M stage						
M0	166	113	0.184	202	198	0.727
M1	9	15		4	3	
N stage						
N0	175	141	0.006	163	147	0.007
N1	36	57		49	77	
N2	28	40		21	19	
N3	0	2		5	0	
T stage						
T1	90	74	0.501	60	48	0.343
T2	124	136		143	143	
T3	22	23		31	39	
T4	8	10		9	14	

**Table 2:** Correlation analysis of SFTPB in AD.

		SFTPB	EGFR	KRAS	TP53	ALK	BRAF	Age
SFTPB	R	1	−0.140	−0.076	0.128	0.044	−0.177	0.028
	P		0.002	0.090	0.004	0.332	<0.001	0.533
EGFR	R	−0.140	1	0.011	−0.123	−0.024	0.061	−0.001
	P	0.002		0.810	0.007	0.595	0.177	0.983
KRAS	R	−0.076	0.011	1	−0.057	−0.004	0.080	−0.022
	P	0.090	0.810		0.209	0.934	0.078	0.624
TP53	R	0.128	−0.123	−0.057	1	0.067	−0.003	0.072
	P	0.004	0.007	0.209		0.139	0.940	0.112
ALK	R	0.044	−0.024	−0.004	0.067	1	−0.040	−0.048
	P	0.332	0.595	0.934	0.139		0.372	0.291
BRAF	R	−0.177	0.061	0.080	−0.003	−0.040	1	−0.050
	P	<0.001	0.177	0.078	0.940	0.372		0.265
Age	R	0.028	−0.001	−0.022	0.072	−0.048	−0.050	1
	P	0.533	0.983	0.624	0.112	0.291	0.265	

**Table 3:** Correlation analysis of SFTPB in SCC.

		SFTPB	TP53	CDKN2A	SOX2	PIK3CA	NOTCH1	Age
SFTPB	R	1	−0.101	−0.085	−0.176	−0.143	−0.050	0.036
	P		0.026	0.062	<0.001	0.002	0.273	0.431
TP53	R	−0.101	1	−0.011	0.058	0.050	0.196	−0.045
	P	0.026		0.810	0.200	0.273	<0.001	0.326
CDKN2A	R	−0.085	−0.011	1	−0.004	−0.014	−0.074	−0.012
	P	0.062	0.810		0.929	0.761	0.104	0.788
SOX2	R	−0.176	0.058	−0.004	1	0.528	0.004	−0.116
	P	<0.001	0.200	0.929		<0.001	0.933	0.011
PIK3CA	R	−0.143	0.050	−0.014	0.528	1	−0.032	−0.025
	P	0.002	0.273	0.761	<0.001		0.482	0.584
NOTCH1	R	−0.050	0.196	−0.074	0.004	−0.032	1	−0.012
	P	0.273	<0.001	0.104	0.933	0.482		0.785
Age	R	0.036	−0.045	−0.012	−0.116	−0.025	−0.012	1
	P	0.431	0.326	0.788	0.011	0.584	0.785	

**Table 4:** Multivariate Cox regression analysis for overall survival according to SFTPB expression.

Variable	AD HR (95% CI)	*p*-Value	SCC HR (95% CI)	*p*-Value
Age	1.011 (0.996–1.027)	0.148	1.017 (1.001–1.035)	0.042
Male vs. Female	1.104 (0.822–1.481)	0.511	1.239 (0.902–1.702)	0.186
Stage III–IV vs. I–II	2.247 (1.641–3.077)	<0.001	1.574 (1.143–2.168)	0.005
SFTPB High vs. Low	0.551 (0.405–0.748)	<0.001	1.347 (1.028–1.767)	0.031

## Data Availability

The data presented in this study are available on request from the corresponding author.
